# Macrophage Tropism and Cytopathicity of HIV-1 Variants Isolated Sequentially from a Long-Term Survivor Infected with *nef*-Deleted Virus

**DOI:** 10.2174/1874285800701010001

**Published:** 2007-06-25

**Authors:** Paul R Gorry, Dale A McPhee, Steven L Wesselingh, Melissa J Churchill

**Affiliations:** 1 Macfarlane Burnet Institute for Medical Research and Public Health, Melbourne, Victoria, Australia; 2 Department of Medicine, Monash University, Melbourne, Victoria, Australia; 3 Department of Microbiology and Immunology, University of Melbourne, Melbourne, Victoria, Australia; 4 National Serology Reference Laboratory, Fitzroy, Victoria, Australia; 5 St. Vincent’s Institute, Fitzroy, Victoria, Australia

## Abstract

Long-term survival of human immunodeficiency virus type 1 (HIV-1) infection has been noted in rare cohorts of individuals infected with *nef*-deleted virus. Enhanced macrophage tropism and cytopathicity contribute to pathogenicity of wild type HIV-1. To better understand the pathogenesis of *nef*-deleted HIV-1, we analyzed the replication capacity and macrophage cytopathicity of *nef*-deleted HIV-1 isolated sequentially from a long-term survivor during progression to AIDS (n=6 isolates). Compared with controls, all *nef*-deleted viruses replicated to low levels in peripheral blood mononu-clear cells and monocyte-derived macrophages (MDM). One *nef*-deleted virus that was isolated on the development of AIDS caused high levels of syncytia in MDM similar to control viruses, but five viruses isolated from earlier times prior to AIDS onset caused only minimal cytopathicity. Together, these results suggest that enhanced cytopathicity of *nef*-deleted HIV-1 for MDM can occur independently of replication capacity, and may contribute to the pathogenesis of *nef*-deleted HIV-1 infection.

## INTRODUCTION

Infection with human immunodeficiency virus type 1 (HIV-1) causes depletion of CD4+ T-cells, and without highly active antiretroviral therapy (HAART) results in acquired immunodeficiency syndrome (AIDS). Left untreated, HIV-1 results in progression to AIDS in the majority of infected subjects, but a small proportion may progress at a significantly slower rate, or may be able to completely control HIV-1 infection and lack any evidence of HIV-1 progression (reviewed in [1]). These subjects are referred to as slow progressors (SP) or long-term nonprogressors (LTNP), respectively. Collectively, SP and LTNP are grouped as long-term survivors (LTS) of HIV-1 infection.

Numerous viral and host factors have been shown to affect the rate of HIV-1 disease progression (reviewed in [1–3]). Viral genetic factors shown to affect HIV-1 progression include mutations in the HIV-1 *nef*, *gag*, *rev*, *vif*, *vpr*, *vpu* and *env* genes. Host genetic factors linked to a delay in the onset of AIDS and prolonged survival include the CCR5 Δ32 mutation, CCR2-V64I polymorphism, and certain HLA haplotypes. The *nef* gene is a major determinant of virulence in primate lentiviruses. Mutations in *nef* attenuate replication capacity and pathogenicity of simian immunodeficiency virus [4–9], and long-term survival of HIV-1 infection has been noted in rare cases of infection with *nef*-defective HIV-1 [10–15].

The largest described cohort of LTS infected with *nef*-defective HIV-1 is the Sydney blood bank cohort (SBBC), which consists of eight individuals who became infected with HIV-1 *via* contaminated blood products obtained from a common donor between 1981 and 1984 [16, 17]. Viral attenuation has been attributed to common deletions in the *nef* and long-terminal repeat regions of the HIV-1 genome [18, 10]. Thus, the SBBC provides an unprecedented opportunity to study the pathogenicity of *nef*-deleted HIV-1 variants in a naturally occurring, human setting.

HIV-1 enters cells *via* the sequential interaction of the Envelope glycoproteins (Env) with the primary CD4 receptor, and then a coreceptor, either CCR5 or CXCR4 (reviewed in [19]). CCR5 dependent (R5) viruses predominate at earlier, asymptomatic stages of HIV-1 infection whereas viruses that have acquired the ability to use CXCR4 instead of- or in addition to CCR5 for cellular entry (referred to as X4 or R5X4 viruses, respectively) emerge at later stages of infection in a significant proportion of individuals (reviewed in [20]). However, most individuals progress to AIDS whilst harbouring only R5 HIV-1 variants (reviewed in [21]). The tropism of HIV-1 is largely determined by coreceptor preference; entry of HIV-1 into macrophage lineage cells is usually mediated by CCR5, although certain X4 and R5X4 strains can enter macrophages efficiently *via* CXCR4 [21]. However, not all R5 HIV-1 isolates are macrophage tropic (M-tropic). In fact, acquisition of M-tropism by HIV-1 during the course of HIV-1 infection contributes to disease progression, irrespective of the coreceptor specificity of the HIV-1 strain [22, 21, 23–25]. Macrophages are also a significant viral reservoir **in vivo** [26, 27], and are a significant source of sustained high level viremia at late stages of infection when virtually all CD4+ T-cells are depleted [28, 29]. Furthermore, the cytopathic effects of HIV-1 infected macrophages are visible microscopically in certain tissues as multinucleated giant cells, and correlate with organ-specific HIV-1 disease; the best characterized of these being HIV-1 encephalitis [30]. Thus, the ability of HIV-1 to replicate and cause cytopathic effects in macrophages contributes significantly to the pathogenesis of HIV-1 infection.

Whether enhanced M-tropism and enhanced macrophage cytopathicity are properties of *nef*-deleted HIV-1 strains isolated from subjects who experienced slowly progressive infection is unknown. To better understand M-tropism and macrophage cytopathicity of *nef*-deleted viruses, and to determine whether these properties are linked to pathogenicity of *nef*-deleted HIV-1, we characterized 6 sequentially isolated *nef*-deleted HIV-1 variants from the SBBC “donor”, subject D36, during progression to AIDS. We found that all 6 *nef*-deleted viruses replicated to low levels in peripheral blood mononuclear cells (PBMC) and monocyte-derived macrophages (MDM), with equivalent efficiency. However, only one *nef*-deleted virus, which was isolated during AIDS, caused high levels of syncytia in MDM that was similar to highly cytopathic control viruses. Five *nef*-deleted viruses isolated from earlier times prior to the development of AIDS caused only minimal cytopathicity. Thus, enhanced cyto-pathicity of *nef*-deleted HIV-1 for MDM can occur without enhancement of M-tropism, and may contribute to the pathogenesis of *nef*-deleted HIV-1 infection in D36.

## MATERIALS AND METHODOLOGY

**Virus Isolates:** Primary viruses D36 II, D36 V, D36 VIII, D36 IX, D36 X and D36 XI have been described in detail previously [31], and were isolated from blood of subject D36 by coculture with CD8-depleted PBMC according to published methods [22, 32]. Characteristics of the HIV-1 isolates are summarized in Table 1. The dates when blood was drawn for HIV-1 isolation were May 1995, July 1996, May 1997, December 1997, July 1998 and January 1999, respectively. Blood was taken in accordance with guidelines endorsed by the Australian Red Cross Blood Service human ethics committee. Analysis of the *nef* and long terminal repeat sequence demonstrated gross deletion mutations in both regions, which are characteristic of SBBC HIV-1 isolates [31, 18, 10]. Analysis of coreceptor usage showed that all the primary isolates from D36 used here were R5X4 [31]. Stocks of the R5 HIV-1 ADA virus [33] were prepared from supernatants of infected PBMC as described previously [22]. Stocks of the X4 HIV-1 NL4-3 and R5X4 HIV-1 89.6 viruses [34, 35] were produced by transfection of 293T cells with proviral plasmid DNA by the calcium phosphate method [36].

**Cells:** PBMC were purified from blood of healthy HIV-1-negative donors, stimulated with 5 μg of phytohemaggluti-nin (PHA) (Sigma, St. Louis, MO) per ml for 3 days, and cultured in RPMI 1640 medium supplemented with 10% (vol/vol) fetal calf serum (FCS), 100 μg of penicillin and streptomycin per ml, and 20 U of interleukin-2 (IL-2) (Roche, Basel, Switzerland) per ml. MDM were purified from PBMC by plastic adherence and cultured for 5 days in RPMI 1640 medium supplemented with 10% (vol/vol) human AB+ serum, 100 μg of penicillin and streptomycin per ml, and 12.5 ng of macrophage colony-stimulating factor (M-CSF) per ml.

**HIV-1 Replication Kinetics:** Five hundred thousand PHA-activated PBMC were infected in 48-well tissue culture plates by incubation with 1 x 10^6^ ^33^P cpm HIV-1 reverse transcriptase (RT) units of virus supernatant in a volume of 250 μl for 3 h at 37°C, as described previously [31, 23]. Virus was then removed, and PBMC were washed 3 times with phosphate-buffered saline (PBS) and cultured in medium containing 20 U of IL-2 per ml for 28 days. Fifty percent medium changes were performed twice weekly, and supernatants were tested for HIV-1 replication by RT assays, as described previously [36]. MDM were isolated from PBMC by plastic adherence and allowed to mature for 5 days prior to infection, as described previously [22]. At approximately 90% confluence in 48-well tissue culture plates, virus equivalent to 1 x 10^6^ ^33^P cpm RT units in a volume of 250 μl was allowed to adsorb to the cell monolayers for 3 h at 37°C. Virus was then removed, and cells were rinsed 3 times with PBS prior to addition of 500 μl of culture medium. Fifty percent medium changes were performed twice weekly for 28 days, and supernatants were tested for HIV-1 replication by RT assays.

**Analysis of Syncytium Formation in MDM:** Cultures of HIV-1-infected MDM were analyzed for syncytium formation at days 7, 11, 14, and 18 post-infection using an Eclipse TE 300 inverted microscope (Nikon, Osaka, Japan). Syncytia were counted by visual inspection and scored as +/- (occasional), + (low), ++ (moderate), or +++ (extensive), as described previously [37]. Photographs were taken at a final magnification of x400.

## RESULTS

**Primary Isolates, and Clinical Characteristics of the Study Subject:** Subject D36 is the SBBC common donor, and has been described in detail previously [31, 17]. Briefly, D36 was infected with HIV-1 sexually in December, 1980. For 14 years without antiretroviral therapy D36 had a stable CD4+ T-cell count, but after April 1994 the subject experienced CD4+ T-cell loss until a diagnosis of HIV-associated dementia (HIVD) was made in December 1998. HIVD was the subjects first and only AIDS defining illness, and coincided with the CD4+ T-cell count falling below 200 cells/μl and plasma viral load steadily increasing to approximately 20,000 RNA copies/ml. At HIVD diagnosis, CSF viral load was measured at >750,000 RNA copies/ml. Following commencement of HAART in January 1999 [31, 38] both plasma and CSF HIV-1 RNA were suppressed to below detectable levels. D36 subsequently experienced neurological improvement and CD4+ T-cell recovery to approximately 700 cells/μl in August 2006. D36 remains clinically well. Thus, although infected with an attenuated, *nef*-deleted strain of HIV-1, D36 can be classified as a SP.

We previously characterized HIV-1 viruses isolated sequentially from D36 over a three year period leading up to HIVD, which follow the CD4+ T-cell loss in this subject. The known phenotypic characteristics of these isolates, laboratory studies corresponding to the times when the blood samples were taken for HIV-1 isolation, and clinical characteristics of the study subject are summarized in Table 1. All isolates were of R5X4 phenotype, similar to HIV-1 89.6. In contrast, HIV-1 ADA and HIV-1 NL4-3 were restricted to use of CCR5 or CXCR4 for entry, respectively (data not shown). Thus, the D36 viruses studied here maintained an R5X4 phenotype over the three year interval.

**Fig. (1). Replication kinetics in PBMC:**
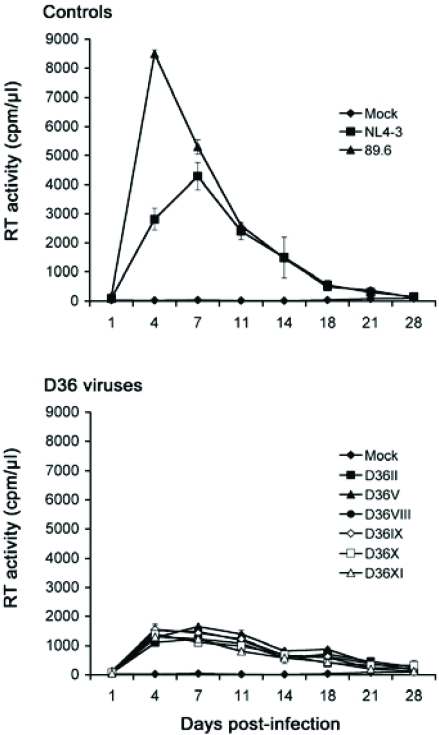
PBMC were infected with equivalent amounts of each virus, as described in Materials and Methodology, and cultured for 28 days. Mock infected cells were treated with culture medium alone. Virus production in culture supernatants was measured by RT assays. Values shown are means from duplicate infections. Error bars represent standard deviations. Results are representative of two independent experiments using cells obtained from different donors, which gave similar results.

**Replication in PBMC:** We first examined the capacity of the primary, *nef*-deleted HIV-1 viruses isolated from D36 to replicate in PBMC compared to control viruses with intact *nef* genes (Fig. 1). The R5X4 89.6 and X4 NL4-3 viruses replicated rapidly to high levels, although 89.6 replicated to higher levels than NL4-3 and with more rapid replication kinetics, peaking at day 4 post-infection. There was no variation between the replication kinetics and levels of peak virus replication attained by the D36 primary isolates. The D36 viruses reached peak levels of virus replication at 4 to 7 days post-infection. However, peak levels of replication attained by the D36 viruses were approximately 6-fold and 3-fold lower than those achieved by 89.6 and NL4-3, respectively. Thus, compared to control viruses the *nef*-deleted D36 viruses have attenuated but similar replication capacity in PBMC.

**Fig. (2). Replication kinetics in MDM:**
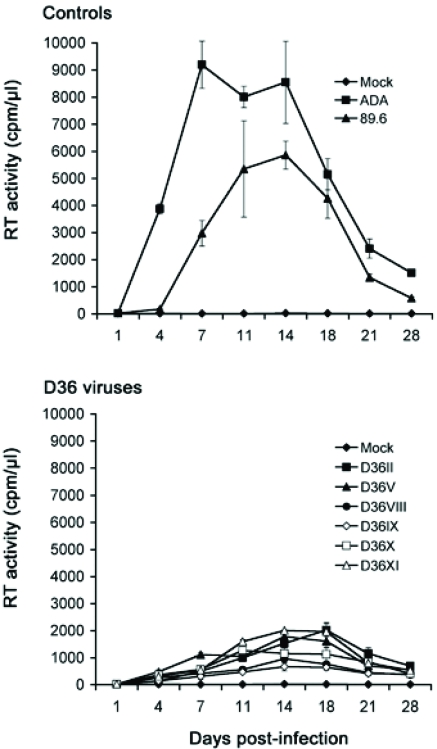
MDM were infected with equivalent amounts of each virus, as described in Materials and Methodology, and cultured for 28 days. Mock infected cells were treated with culture medium alone. Virus production in culture supernatants was measured by RT assays. Values shown are means from duplicate infections. Error bars represent standard deviations. Results are representative of two independent experiments using cells obtained from different donors, which gave similar results.

**Replication in MDM:** Although all the D36 isolates are phenotypically R5X4 (Table 1 and [31]), we recently showed that certain R5X4 viruses isolated from blood of a subject with HIVD were highly M-tropic, and that efficient macrophage entry by these viruses occurred *via* CXCR4 [22]. These studies raised the possibility that enhanced tropism of R5X4 viruses for macrophages may contribute to neurovirulence. It is presently unknown whether *nef*-deleted R5X4 viruses harbored by D36 are M-tropic, or whether enhancement of M-tropism by these viruses contributed to disease progression in this subject. Thus, we next examined the capacity of the sequential D36 viruses to replicate in MDM compared to two well characterized M-tropic control viruses, 89.6 and ADA (Fig. 2).

The R5 ADA virus replicated rapidly to high levels, attaining peak levels of virus replication at day 7 post-infection. The R5X4 89.6 virus also replicated to high levels, but had delayed replication kinetics compared to ADA, reaching peak levels of virus replication at day 14 post-infection. All the R5X4 D36 viruses were able to replicate to detectable levels in MDM, and had similar replication kinetics to the R5X4 89.6 control virus peaking at days 14 to 18 post-infection, but attained peak levels of virus replication that were approximately 3-fold lower than 89.6. Although there was some variation in replication capacity among the D36 viruses, there was no appreciable difference in replication capacity between viruses when tested across multiple MDM donors (data not shown). These data indicate that the D36 viruses have reduced infectivity for MDM compared to ADA and 89.6, and that infectivity for MDM was not enhanced when isolates were sequentially isolated during HIV-1 progression in D36.

**Fig. (3). Syncytium formation in MDM induced by nef-deleted viruses:**
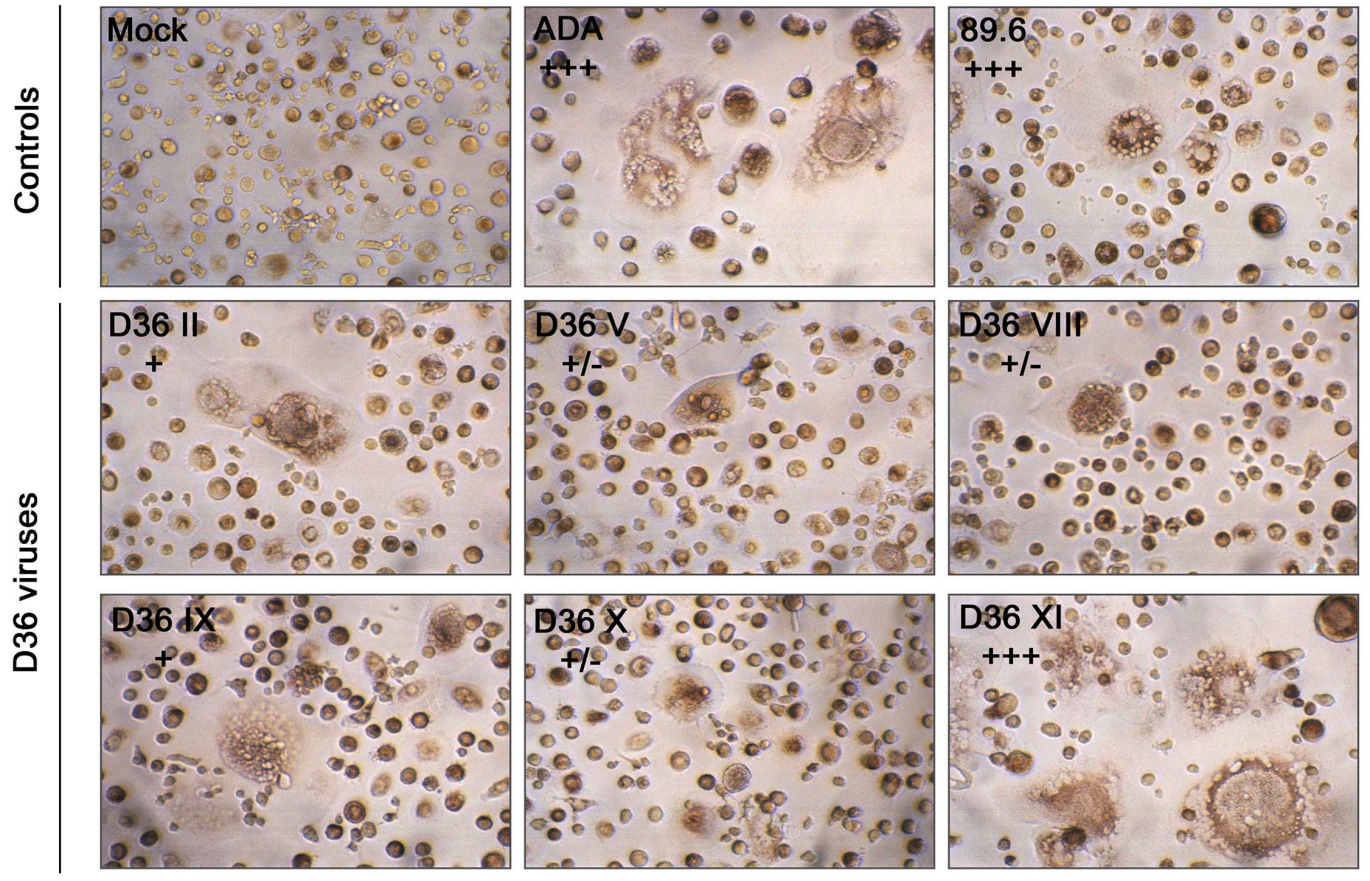
MDM were infected with equivalent amounts of each virus, as described in Materials and Methodology. Mock-infected MDM were treated with culture medium alone. Syncytia formation was documented at day 11 (ADA), 14 (89.6) or 18 (D36 viruses) post-infection. Syncytia were counted manually and scored as +/-, occasional syncytia; +, low frequency of syncytia, occurring in <5% of cells; ++, moderate frequency of syncytia, occurring in 5 to 50% of cells; or +++, extensive syncytia, occurring in >50% of cells, as described previously [37]. Results are representative of two independent experiments using cells obtained from different donors, which gave similar results. Photographs are at a final magnification of x400.

**Cythopathicity in Macrophages:** Primary HIV-1 isolates may have vastly differing cytopathic effects in MDM that do not necessarily correlate with replication capacity [37], suggesting that MDM cytopathicity may be dictated by factors other than those that permit efficient viral entry. However, the ability of HIV-1 to be cytopathic in MDM may be associated with HIV-1 pathogenesis. For example, the ability of neurotropic HIV-1 isolates to induce syncytia in MDM cultures is closely associated with the presence of multinucleated giant cells in brain [37], which is a neuropa-thological hallmark of HIVD. Cultures of MDM infected with the sequential D36 viruses, or the R5 ADA or R5X4 89.6 control viruses, were examined for syncytia formation (Fig. 3). ADA and 89.6 induced syncytia in >90% of cells by day 11 or 14 post-infection, respectively. D36 II, D36 V, D36 VIII, D36 IX and D36 X viruses, which were all isolated prior to the development of AIDS, induced syncytia in <5% of cells throughout the 28 days of infection. In contrast, D36 XI, which was isolated on development of AIDS, induced syncytia in >90% of cells by day 18 post-infection, despite replicating only to low levels in these cultures (Fig. 2). Thus, high levels of cytopathicity were generated in MDM infected with D36 XI virus, similar to the highly cy-topathic control viruses.

## DISCUSSION AND CONCLUSION

In this study, we characterized a panel of HIV-1 viruses sequentially isolated from a SP infected with *nef*-deleted virus, that tracked CD4+ T-cell loss leading to the development of AIDS in this subject. The replication capacity of the sequential isolates in PBMC and MDM was not enhanced during progression of HIV-1 infection, but significant cyto-pathicity in MDM was evident only in cells infected with virus isolated on development of AIDS. These results suggest that *nef*-deleted HIV-1 strains may evolve during HIV-1 progression and increase their cytopathic potential without necessarily replicating more efficiently in the host. This would explain the CD4+ T-cell loss and progression to AIDS in D36 despite only relatively low plasma HIV-1 RNA levels.

Whilst the SBBC recipient members persistently harbor *nef*-deleted HIV-1 with an R5 phenotype [32], the viruses isolated from D36 used in this study maintained an R5X4 phenotype over the 3 year period [31]. This suggests that virus harbored by D36 evolved from an R5 to R5X4 phenotype prior to 1995. The emergence of R5X4 HIV-1 is typically associated with rapid progression to AIDS [39–43]. However, D36 harbored R5X4 HIV-1 for at least 3 years without antiretroviral therapy and experienced only slow progression of HIV-1 infection [31]. Thus, *nef*-deleted HIV-1 in an attenuated infection may undergo a coreceptor switch from R5 to R5X4 phenotype without subsequent rapid progression to AIDS. Nonetheless, it remains uncertain as to whether D36 would have experienced HIV-1 progression at all had virus harbored by this subject not undergone a core-ceptor switch.

Although we demonstrate increased macrophage cyto-pathicity by virus isolated from blood of D36 during HIVD, whether the circulating strain of virus contributed to neuro-pathogenesis of HIV-1 infection in D36, which was the subject’s only AIDS defining illness, remains unclear. CSF HIV-1 RNA levels were considerably higher than plasma HIV-1 RNA levels during HIVD (>750,000 copies/ml versus approximately 20,000 copies/ml) [31]. A CSF-derived virus was never able to be successfully isolated despite repeated attempts, but analysis of the HIV-1 Env V3 sequence in CSF demonstrated an R5-like sequence [31], suggesting that HIV-1 present in CSF of D36 was most likely an R5 virus. This analysis showed compartmentalized evolution of *nef*-deleted HIV-1 in D36, raising the possibility that the direct neuropa-thogenic effects were caused by distinct pathogenic features of the CSF virus not present in the blood-derived isolates. HIVD typically occurs late in HIV-1 infection, after the onset of immunodeficiency [44]. Therefore, it is plausible that evolution toward a more cytopathic *nef*-deleted virus in blood contributed to neuropathogenesis in D36 by lowering the immune threshold for the neuropathogenic manifestations of HIV-1 infection to become evident.

The molecular determinants underlying increased macrophage cytopathicity by the D36 XI isolate are unclear, but most likely map to the HIV-1 Env (reviewed in [21]). The Env glycoproteins are the principal determinants of cytotox-icity in an infected cell [45]. Furthermore, studies in macaques infected with chimeric simian-HIV (SHIV) viruses showed distinct changes in Env associated with enhanced pathogenicity **in vivo**, which in part resulted from increased Env-coreceptor binding [46]. Therefore, one possibility is that R5X4 Env glycoproteins in D36 isolates evolved to variants able to interact more efficiently with cellular receptors, thus increasing their cytopathic potential. This hypothesis is supported by previous studies which analyzed the ability of D36 II and D36 XI viruses to cause CD4+ T-cell cyto-pathicity in *ex vivo* human lymphoid cell cultures [47]. This study found that D36 XI was significantly more potent in depleting CD4+ T-cells from these cultures than D36 II, which resulted from an increased ability of D36 XI to use CXCR4 as a coreceptor for HIV-1 entry. Thus, increased macrophage cytopathicity by D36 XI is most likely due to intrinsic pathogenic features of the Env that increase fu-sogenicity, similar to that which has been observed by neu-rotropic R5 and R5X4 viruses [37, 48, 49]. This idea is consistent with previous studies that linked increased Env-mediated fusion to pathogenicity of *nef*-deleted SIV [50]. Further studies are required to elucidate the molecular determinants of D36 Env that are associated with increased fusogenicity.

In summary, we demonstrate increased macrophage cy-topathicity by *nef*-deleted R5X4 HIV-1 isolated from blood of a SP during progressive HIV-1 infection. To our knowledge, this is the first report to demonstrate enhanced macrophage cytopathicity by a *nef*-deleted HIV-1 variant associated with AIDS. The molecular determinants underlying increased macrophage cytopathicity by this HIV-1 variant remain unclear, but involve mechanism(s) distinct from those that govern replication capacity *per se*. Increased macrophage cytopathicity is likely to involve changes in the Env glycoproteins, which also contribute to CD4+ T-cell loss in D36.

## Figures and Tables

**Table 1:** HIV-1 Isolates, Coreceptor Usage, Clinical History of the Study Subject and Corresponding Laboratory Studies

HIV-1 Isolate	Date of Blood Sample	CD4+ T-Cell Count (Cells/μl)	Plasma Viral Load (RNA Copies/ml)	Coreceptor Usage of HIV-1 Isolate*	Clinical History*
D36 II	5/1995	NT	1400	R5X4	Diagnosed with HIVD 12/1998
D36 V	7/1996	414	2600	R5X4	
D36 VIII	5/1997	540	4000	R5X4	ABC, AZT, NVP (1/1999-9/2004)
D36 IX	12/1997	390	7500	R5X4	
D36 X	7/1998	325	NT	R5X4	ABC, NVP, 3TC (9/2004-present)
D36 XI	1/1999	160	9900	R5X4	

CD4+ T-cells were measured by flow cytometry. Plasma HIV-1 RNA was measured by COBAS Amplicor HIV-1 Monitor Version 1.0 (Roche Molecular Diagnostic Systems, Branchburg, N.J.). NT, not tested; HIVD, HIV-associated dementia; ABC, abavavir; AZT, zidovudine; NVP, nevirapine; 3TC, lamivudine. *These results have been reported previously [31, 18].
